# Re-introduction of Griffon Vulture (*Gyps fulvus*) in the Eastern Balkan Mountains, Bulgaria – completion of the establishment phase 2010-2020

**DOI:** 10.3897/BDJ.9.e66363

**Published:** 2021-04-20

**Authors:** Elena Kmetova–Biro, Emilian Stoynov, Ivelin Ivanov, Hristo Peshev, Simeon Marin, Lachezar Bonchev, Iliyan P. Stoev, George Stoyanov, Zlatka Nikolova, Nadya Vangelova, Dimitar Parvanov, Atanas Grozdanov

**Affiliations:** 1 Green Balkans – www.greenbalkans.org, Stara Zagora, 9 Stara Planina Str., Bulgaria Green Balkans – www.greenbalkans.org Stara Zagora, 9 Stara Planina Str. Bulgaria; 2 Department of Environmental Sciences and Policy, Central European University, Vienna, Austria Department of Environmental Sciences and Policy, Central European University Vienna Austria; 3 Fund for Wild Flora & Fauna, 49 Ivan Mikhaylov Str., office 327, P.O.Box 78, www.fwff.org, pirin@fwff.org, Blagoevgrad, Bulgaria Fund for Wild Flora & Fauna, 49 Ivan Mikhaylov Str., office 327, P.O.Box 78, www.fwff.org, pirin@fwff.org Blagoevgrad Bulgaria; 4 Department of Geography, Ecology and Environmental Protection, Faculty of Mathematics and Natural Sciences, South-West University “Neofit Rilski”, Blagoevgrad, Bulgaria Department of Geography, Ecology and Environmental Protection, Faculty of Mathematics and Natural Sciences, South-West University “Neofit Rilski” Blagoevgrad Bulgaria; 5 Birds of Prey Protection Society, 23 Golyam Bratan Str., www.bpps.org, Sofia, Bulgaria Birds of Prey Protection Society, 23 Golyam Bratan Str., www.bpps.org Sofia Bulgaria; 6 Nadezhda Women’s Health Hospital, 3 “Blaga vest” Street, Sofia, Bulgaria Nadezhda Women’s Health Hospital, 3 “Blaga vest” Street Sofia Bulgaria; 7 Department of Zoology and Anthropology, Faculty of Biology, Sofia University “St. Kliment Ohridski”, 8 Dragan Tsankov Blvd, zootribe@gmail.com, Sofia, Bulgaria Department of Zoology and Anthropology, Faculty of Biology, Sofia University “St. Kliment Ohridski”, 8 Dragan Tsankov Blvd, zootribe@gmail.com Sofia Bulgaria

**Keywords:** Griffon Vulture, re-introduction, establishment, release, feeding site, home range, breeding performance, conservation management, Kotlenska planina SPA, Sinite Kamani Nature Park, source-sink, GPS tracking

## Abstract

The current study presents and analyses the results from the recently completed 11-year-establishment phase, following the start of the local re-introduction of the Griffon Vulture (*Gyps fulvus*) in Kotlenska Planina SPA and Sinite Kamani Nature Park in the Eastern Balkan Mountains of Bulgaria in the period 2010-2020. As a result of the re-introduction efforts and release of 153 individuals, the Griffon Vulture has been successfully reproducing again in the Eastern Balkan Mountains since 2016, after more than 40-50 years of absence. At 2020, the local population consists of some 80 local and up to 80-115 birds, together with sojourn individuals. Amongst them, 23-25 breeding pairs, located in five different colonies and two more frequently used roosting sites. The current average productivity remains relatively low: 0.41 fledglings/territorial pair and fledging success of 0.61 fledglings/breeding pair between 2016 and 2020, but shows a trend to increase with time and the growing experience of the young locally re-introduced population. The mortality confirmed between 2010-2021 accounts for 33%, mostly due to electrocution as a post-release effect in the first six months following their release. Our data show that the newly established population in the Eastern Balkan Mountains mostly forages on feeding sites, having a comparatively small 95% home range: 281.88 ± 91 km^2^ and 50% core area: 6.6 ± 2.28 km^2^ (range 4.7–8.5 km^2^). We, therefore, consider the establishment phase of the re-introduction of Griffon Vulture in this particular site as successfully completed, but management should continue. Furthermore, the area of the Eastern Balkan Mountains can currently be regarded as a "source" for the species within the source-sink population regulation concept in the national and Balkan context.

## Introduction

The Griffon Vulture *Gyps fulvus* (Hablizl, 1783) is a Western Palearctic cliff-nesting social obligate scavenger and one of the largest birds of prey in Europe (Cramp and Simmons 1980). It used to be widespread and numerous in Bulgaria up to the 1940s (Patev P. 1950), but became less frequently observed and likely became extinct as a breeding species around the 1970s (Baumgart 1974, Demerdzhiev et al. 2007). The Eastern Balkan Mountains and the area of the town of Kotel were amongst the last sites where breeding of the species was reported (Donchev 1974).

A small breeding colony (1-4 pairs) was discovered in southern Bulgaria along the border with Greece in the Eastern Rhodopes in 1978 (Michev et al. 1980, Yankov and Profirov 1991) and later, due to a set of conservation measures, the population reached some 70 breeding pairs in 2014 (Demerdzhiev et al. 2014, Dobrev and Stoychev 2014). Despite such a promising recovery, the breeding range remained isolated within a 20-30 km stretch along the Arda River (UTM, LG71 and MG01) with no expansion out of the Eastern Rhodopes towards other parts of the country within the historic range of the species (Demerdzhiev et al. 2007, Stoynov et al. 2018b).

Considering the unique ecological role and ecosystem services provided by vultures and having the good example for successful re-introduction of the species in some historic sites in France (Terrasse et al. 2004), an international initiative called the Balkan Vultures Action Plan was developed in 2002 (Tewes et al. 2004) to review its former distribution and contemporary conditions for restoration. One of the aims of the plan was to secure the long-term survival of the Griffon Vulture population in Bulgaria, increasing its number and expanding its breeding range through strategic re-establishment of colonies in former breeding sites. An important component in the strategy was also to use the recovery of the Griffon Vulture - listed as "least concern" (BirdLife International 2002), as a proxy species for the future restoration of Cinereous (*Aegypius monachus*) and Bearded (*Gypaetus babatus*) vultures, both considered extinct in Bulgaria (Botev 1985, Golemanski 2011) and listed as "near-threatened" globally and the Bearded Vulture also as "endangered" in the European Red Data List (BirdLife International 2002).

The plan was developed to increase the national population and re-establish some of the historic ranges, considering its importance in a Balkan regional context, through releases of Griffon Vultures found in distress and rehabilitated, as well as captive-bred individuals, translocated from Spain and France in strategically-chosen sites throughout Bulgaria. In the national context, the release of individuals in the Balkan Mountains is considered as re-stocking of the Bulgarian population of Griffon Vulture in line with the IUCN Guidelines for Reintroduction (IUCN/SSC 2013). At the same time, each re-establishment initiative is locally referred as local re-introduction. We use the term “re-introduction” as defined by IUCN (IUCN 1987) to identify an intentional movement of an organism into a part of its native range from which it has disappeared or became extirpated in historic times. Re-introduction is considered successful when the species is “established”, meaning that survival and successful breeding of both the founder individuals and their offspring is confirmed (Seddon et al. 2012). The “establishment phase of a re-introduction” refers to the period, when the population is susceptible to particular threats that will disappear once the population survives this phase (Armstrong and Seddon 2008).

**Figure 1.**
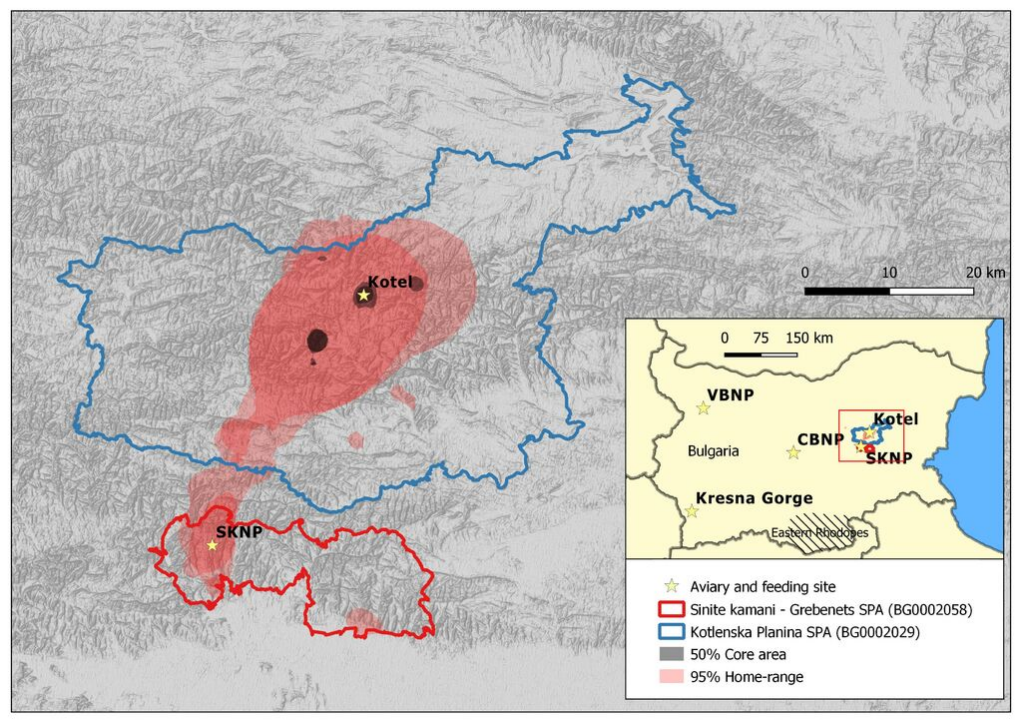
The inserted locator map indicates the location of the Griffon Vulture autochthonous population in the Eastern Rhodopes, southern Bulgaria (shaded), as compared to the four re-introduction areas with their respecitve release sites (i.e. Kotel and SKNP in EBM shown within the red square, CBNP, VBNP and Kresna Gorge) throughout Bulgaria (all release sites are indicated with yellow stars). The close-up map presents the release area in EBM with Kotlenska Planina SPA (blue polygon) and Sinite Kamani - Grebenets SPA (red polygon). The pink shaded area represents 95% of the home-range of the Griffon Vulture in EBM in 2020, while the black shaded spots depict 50% of the core area. Data calculated on the basis of GPS-tracked individuals.

Following years of preparatory work and feasibility studies, a practical local re-introduction of Griffon Vulture was started in 2010 by three Bulgarian nature-conservation NGOs. In line with the preliminary studies carried out, simultaneous releases of individuals of the species began at four sites along the Balkan Mountains of Bulgaria: Vrachanski Balkan Nature Park (hereafter VBNP) (UTM, FN99), Central Balkan National Park (CBNP) (UTM, LH32), Sinite Kamani Nature Park (SKNP) (UTM, MH43) and Kotlenska Planina (UTM, MH65; a few birds were released already in 2009) (Stoev et al. 2016, Stoyanov et al. 2016, Yankov et al. 2016) and Kresna Gorge (UTM, FM73) (Peshev et al. 2015) (all presented on Fig. 1)

The stages and milestones in the establishment phase of the local re-introductions, foreseen in the feasibility studies, were as follows:

1. Establishment of a local non-breeding nucleus and permanent presence of minimum eight individuals year-round at the particular release site;
2. Post-release effects (Armstrong and Reynolds 2012) no longer playing a role and first successful breeding by founder individuals recorded;
3. Establishment phase of Griffon Vulture local re-introduction considered complete, once the local breeding nucleus is producing about 10 offspring a year and locally hatched and raised individuals start reproducing on their own.

The current publication reports the results of the local re-introduction programme of Griffon Vulture in the Sinite Kamani Nature Park and the Kotlenska Planina SPA from the onset of the releases in 2010 until 2020. Due to the close proximity of the two release sites (< 20 km line of sight) and the common movement patterns and behaviour of the vultures released so far, the two sites are reviewed as a single site, referred to as “Eastern Balkan Mountains” (hereafter EBM).

## Material and methods

### Release and management techniques

Acclimatisation aviaries and feeding sites (also known as "supplementary feeding sites" or "vulture restaurants") were built and employed in two areas, namely the Sinite Kamani Nature Park (UTM, MH43) near the town of Sliven and Kotlenska Planina (UTM, MH65) near the town of Kotel. For the period 2007-2020, a total of 153 Griffon Vultures were released in the EBM, as shown in Table 1. Vultures used for releases have been either captive bred or individuals found in distress and rehabilitated (mainly immatures, but also some captive-bred juveniles and few adults), imported from Spain, France and zoos and rehabilitation centres across Europe. They were kept in the aviaries and hard- (by hand) or soft- (opening the cage and exiting on their own when ready) released after 2 to 12 months of stay, in line with the methods developed and applied in the 1980s in Massif Central, France (Terrasse and Choisy 2007).

Following the Griffon Vulture release and adaptation methodology described by Terrasse et al. (2004), food was frequently provided at fenced feeding sites, located in front of the acclimatisation aviaries. Between 2010 and 2020, an average of 170 feedings per year (min. 2-3 times a week) were done per site, providing annually an average total of 30 tonnes of carcasses (mostly pigs, sheep and cattle) and slaughterhouse offal. The frequency and quantity of food provision in EBM gradually increased with time after the start of the releases of the Griffon Vultures, from ca. 15-20 tonnes per year in 2010-2012 to ca. 45-60 tonnes per year in 2017-2020.

### Monitoring technique

For the purpose of monitoring and analyses, the borders of the release area had to be defined. The term "release area", therefore, refers to the two Natura 2000 sites - Kotlenska Planina SPA (BG0002029) and Sinite Kamani-Grebenets SPA (BG0002058) (comprising SKNP) and the territory between them, covering a total area of 2370 km^2^. The combined area is altogether the subject of intensive conservation and management measures aiming at reducing threats (e.g. insulation of power lines, prevention of poisoning) and improving the habitat quality (e.g. nest site reconstruction and optimisation, support of extensive livestock breeding, feeding sites maintenance) for the locally-re-introduced vultures (see map Fig. 1).

In order to ensure individual identification of the Griffon Vultures released within the re-introduction project in EBM, they were all marked with standard metal ornithological rings, PVC colour rings and wing tags with matching inscriptions. The first seven chicks hatched into the wild were also marked with rings and wing-tags prior to fledging and some were tagged with GPS/GSM transmitters to follow their dispersal and survival.

The vultures visiting the two feeding sites and the identified roosting sites were monitored weekly through direct observations and recording of the individual birds present. Additionally, photo traps were also occasionally used at the feeding sites and the footage was analysed on a regular basis, recording the wing-tags of the birds identified, as well as the maximum number of vultures counted (both tagged and non-tagged) on site. All observations were manually entered into a special online storage database with some analytical functions.

Breeding attempts were recorded by surveying all potentially suitable cliffs in the areas of release and in range of 20 km within the two release sites. Cliffs and identified occupied localities were visited a minimum of once a week in the period January – August. Observations were carried out in good weather and visibility, at a distance of 300 to 1300 m from the particular cliff to minimise disturbance, using spotting scopes (30×60 and 20-60×80). For all identified nests, the following information was reported: breeding birds (identified in the majority of cases through their wing-tags and colour rings), nest location coordinates; time of occupation; activity at the time of the observation (nest building, mating, laying, chick rearing etc.). In order to follow the yearly progress and development of the population, the following data were collected: 1. number of occupied nests (all nests occupied by breeding and non-breeding pairs); 2. number of breeding pairs (pairs that were observed incubating); 3. breeding success (fledged juveniles per incubating pair); 4. productivity (fledged juveniles per occupied nest); and 5. hatching success (hatchlings per incubating pair).

For comparative purposes, the current study adopts the definitions and criteria introduced by Demerdzhiev et al. (2014), as follows: a pair was considered formed, but non-breeding, if both birds exhibited attachment to a particular niche (ledge) and did not lay eggs, yet two or more of the following types of behaviour were observed: courtship flights, mutual preening, copulation, nest building and defence of the immediate vicinity of the chosen nest site from conspecifics.

The current study identifies a colony as a cliff, occupied by at least two Griffon Vulture pairs, found at least 1 km away from the nearest other occupied site, as introduced by Demerdzhiev et al. (2014). A hatchling was considered fledgling past the age of 125 days (Cramp and Simmons 1980), while, for the marked chicks in the nest - when seen perched out of the nest or flying in the area.

In the period 2017-2020, several Griffon Vulture individuals of various age groups were tagged with OrniTrack-P33 transmitters (produced by Ornitela, Vilnius, Lithuania). The location of the birds tracked was acquired using a global positioning system (GPS) and transmitted via a public mobile phone network (GSM). If the birds were out of the coverage area of the given network operator, the location data were saved by the device and sent once the transmitter was back within range. The transmitters were set to take GPS fixes every 10 minutes and to transmit the data every 1 to 4 hours and provide information, not only on the GPS location of the bird, but also on elevation (m a.s.l.), speed (km/h) and acceleration (activity), amongst others.

The transmitters were mounted on the patagium, together with a vinyl wing-tag and weighed 33 g or ca. 1% of the body mass (< 3% is recommended for flying birds). A vulnerable attaching element was deliberately used in order to ensure the device falling off after a couple of years. The transmitters were mounted with the necessary precautions and care to minimise the stress for the birds tagged.

The GPS data obtained from such OT-P33 transmiters were used to calculate home ranges and feeding events for two Griffon Vultures tagged in EBM.

The two tracked individuals in EBM were an adult breeding Griffon Vulture (K5M), tagged four years following its release and a juvenile Griffon Vulture (H1) hatched in the area. K5M was tracked between 24.1.2017 and 27.1.2018 for a total of 369 days (a total of 21918 fixes). H1 was tracked between 8.3.2017 and 28.10.2019, for a total of 965 days (a total of 68012 fixes). The data from the two vultures tracked in 2017 were processed for an additional study on the frequency of feedings and food sources utilised in EBM. The latter were divided into two categories – 1. feeding sites (one near Kotel and another one in SKNP); and 2. “wild” feeding (i.e. the vultures found food in the area themselves, without its being placed intentionally or organised by the project team). Each such landing was checked on site by a project team member and "wild" feeding confirmed by finding the eaten carcass. The GPS data of detected landings in the area of the feeding sites were compared against the food provision data sheets and, when it was known that food had been present on site and the bird had stayed for more than 10 minutes (more than one fix at a given location), a "feeding event at the feeding site" was reported.

### Calculations and statistics

Breeding success, fledging success, survival rate, mortality causes and demographic parameters were calculated on the basis of annual averages. Additionally, the drop-out of the released individuals was grouped by time after the release - for example, setting a time buffer of 0-6 and 6-12 and > 12 months in line with Sarrazin et al. (1996) and Sarrazin (2008) to analyse whether recapture/death has occurred as a post-release effect. The survival of the individuals considered as successfully acclimatised was calculated the same way and compared to the natural mortality reported for other populations - such as the ones published by van Beest et al. (2008).

The source/sink population regulation (Pulliam 1988) characteristics of the newly-established meta-population were calculated to assess the value of the locally-re-introduced nucleus of Griffon Vulture in EBM in the national and regional (Balkan Peninsula) context. These calculations were based on the survival and mortality rates of all age groups of wild exogenous (guest-immigrants) individuals and re-introduced birds released > 12 months ago (to exclude post-release effect of drop-out/mortality) compared to the number of young, successfully fledged birds in the area.

The home range of the vultures, released in the EBM, was calculated on the basis of a total of 1305 tracking days of two tagged vultures (see Table 7). A dataset, comprising over 78,000 GPS fixes, was analysed. The home range of each vulture was calculated using the dynamic Brownian Bridge Movement Model (dBBMM) (Kranstauber et al. 2012). Statistics were undertaken with the R free software environment for statistical computing and graphics Version 4.0.3 (R Core Team 2020), using the "adehabitatHR" (v.0.4.18; Calenge 2006, Calenge 2019) and the "Move" (v.4.0.6; Kranstauber 2020) packages. Home range was calculated only for individuals, tracked for at least 100 consecutive days after the release (for re-introduced vultures) or for at least 30 days (for captured indigenous birds). Only locations taken in the interval within 06:00-18:00 UTC+2 were used. The location error was less than 20 m.

A 95% home range was defined as the general individual home range and 50% home range was defined as the core area.

## Results

### Survival, mortality, emigration and immigration

The status of the Griffon Vultures released in the EBM between 2007 and 2020 in December 2020 is presented in Table 2. Our observations show that 34% (n = 52) of the total number of vultures released have attempted breeding in the areas of release and an additional 5% of the birds (n = 7) have settled and attempted breeding in other nearby Griffon Vulture colonies (i.e. Eastern Rhodopes, VBNP, North Macedonia, Kresna Gorge, Messolonghi). A total of 28% (n = 43) of all birds released until now have left the area of release with no further information on their location and status. Despite the fact that they are clearly marked with wing-tags and rings, it is still possible that they are alive and have settled elsewhere, so their number is presented separately. The aggregate mortality confirmed in December 2020 accounts for 33% (n = 51) and two-thirds of it (n = 34) has occurred in the area of release.

A total of 51 vultures of the ones released (n = 153) in the EBM have been confirmed dead in the period 2010-2020. This accounts for a total of 33% of confirmed mortality. The main cause of mortality, responsible for a total of 64% of all confirmed cases, is electrocution on power-lines (n = 33) and a total of 67% of the confirmed mortality cases have been reported within the release area (see Table 3).

The aggregate number of individually identified Griffon Vultures observed in EBM per year has gradually increased from 12 individuals in 2010 to 105 individuals in 2020. At the start of the re-introduction programme in 2010, a total of 92% of the individuals observed in the area were the ones released (it should be noted that shortly after the first releases, wild exogenous guest birds were attracted). This proportion dropped to 43% in 2013, slightly increasing to 54% in 2018 and it is about 48% in 2020. Vultures, originating from the three other release sites: CBNP, VBNP, Kresna Gorge, as well as the autochthonous populations in the Eastern Rhodope Mountains (Bulgaria), Serbia and Croatia, have been identified on site (Table 4). In December 2019, a record-breaking number of 115 Griffon Vultures was counted at one time.

### Breeding performance - number of pairs, colonies

The first breeding attempt at some 60 km to the east of both release sites was accidentally reported in 2012. In the period 2016-2020, a total of 31-33 chicks have been observed to have successfully fledged into the wild (Table 5). The first breeding (including egg laying) attempts were unsuccessful (2012-2015) with the first successful fledging of five offspring reported in 2016 and continuing onwards. Most breeding pairs (n = 16-18) have been reported in 2020, producing a total of 8 to 10 fledglings and showing a continuous positive trend. The average breeding success of the newly-restored Eastern Balkan population accounts for 0.41 fledglings/territorial pair and fledging success of 0.61 fledglings/breeding pair between 2016 and 2020 (Table 5).

The number of territorial pairs has gradually increased from 0 prior 2012 and 1 in 2012-2013 to 23-25 in 2020, distributed in 4-5 colonies as follows: Kotlenska Planina SPA – "Terzievi Porti", "Urushki Skali" (two colonies on the same cliff), "Zlosten", "Orlovite peshteri" and "Orlitsata" - all found from 0.5 to 7 km in different directions from the project release and feeding sites in the area. Additionally, two sites to the east and to the west of the lift line (3-4 km from the release and feeding sites in the area) in the SKNP have been recently occupied, but nesting has still not been confirmed.

### Number of the local population, source/sink balance

In order to assess the source/sink balance characteristics of the newly-established Griffon Vulture population nucleus in EBM, we have reviewed the annual mortality of exogenous vultures on site, extracted from the annual number of successfully-fledged chicks hatched in the area. Table 6 shows that the balance is positive and the number of locally-produced birds exceeds the mortality reported on site.

### Home range estimation

The home range of the two Griffon Vultures tracked in EBM has been calculated as follows: 95% home range: 281.88 ± 91 km^2^ (range 216.85–346.91 km^2^) 50% core area: 6.6 ± 2.28 km^2^ (range 4.7–8.5 km^2^) (Table 7).

### Feeding on vulture restaurants and in the wild

The two vultures tracked in EBM clearly prefer foraging at the feeding sites (80% of the recorded feedings n = 210), while 20% of the feedings (n = 42) were "wild".

## Discussion

### Survival, mortality, emigration and immigration

The overall mortality of 33% of the vultures confirmed dead following their release into EBM for the period 2010-2020 (presented in Table 2) compares to the data published by Sarrazin et al. (1996) for France. Sarrazin et al. (1996) report 27% (n = 59) of the vultures that have been released between 1981 and 1991 in France to have died or been removed from the population, due to emaciation or injuries. At the same time, it should be noted that there are no further observations and data for the survival for a total of 43 or 28% of the vultures released in EBM. It is very likely that a certain proportion of the birds, for which data are missing after their release, have indeed died of starvation, being unable to find the feeding site and/or to adapt to the wild. However, our data clearly show that electrocution is the main cause of death in EBM, causing a total of 64% of the reported mortalities. Most of them occurred along two particular power-lines in close proximity to both of the acclimatisation aviaries and immediate measures for safeguarding the most dangerous sections and particular pylons were enforced. Mortality was mainly observed as a post-release effect during the first-year-adaptation into the wild of the respective individuals and, towards the end of the studied period, the impact of this factor declined. On one hand, this is because of the insulation efforts (authors unpublished data) and, on the other hand, it is due to build-up of flight experience in the surviving individuals, which would less frequently perch on electricity pylons. Table 3 clearly shows that the survival chances of the re-introduced birds significantly increase already after the first six months post-release and become even higher after the first year.

Poisoning, which is otherwise stated as the top reported cause of vulture mortality and decline on the Balkans (Parvanov et al. 2017, Stoynov et al. 2018a), elsewhere in Europe and globally (Botha et al. 2017), has been confirmed in only 2% of the reported mortality cases. It accounts for just a single case, which occurred outside the release area. We, therefore, consider that this threat is only minimal in the re-introduction area and this is a prerequisite for the newly-established population to survive, even if it maintains lower breeding parameters. The rest of the causes of death remain unidentified or random, such as: emaciation, collision with a vehicle, predation etc.

In terms of population dynamics, permanent emigration was occasionally recorded. It was mostly obsreved in birds released as adults and in the very beginning of the re-introduction process, while the local nucleus was not yet fully established and mostly comprised of immature individuals. The surviving emigrants have discovered and settled in existing nearby colonies of the species - Eastern Rhodopes, Bulgaria (n = 5); Tikvesh, North Macedonia (n = 1); Messolonghi, Greece (n = 1); as well as other local re-introduction sites in Bulgaria - VBNP (n = 2) and Kresna Gorge (n = 1). Most of the immature birds, reported as having left the release area, have been confirmed dead after some time (n = 9). As an exceptional case, an adult Griffon Vulture released in SKNP in 2013 was observed in France a few months after its release.

The growing local nucleus of permanently-present vultures (about 80-85 individuals at December 2020) has also attracted more immigrants with time, reaching up to about 50-60 distinguished individuals throughout 2019 and 2020 and increasing the total number of the local group by 25-70% in different periods and occasions. Griffon Vultures tagged elsewhere in the Balkans (Serbia, Croatia, all release and autochthonous sites in Bulgaria) have been frequently observed to visit and sojourn in the EBM. Nesting attempts of "wild exogenous guests" have also been observed (Stoynov et al. 2018b). Vultures tagged in Israel have been frequently reported, yet they are usually birds from the Balkans, tagged during their wintering in the Middle East (O. Hatzofe, pers. comm.).

### Breeding performance - number of pairs, colonies

**Figure 2.**
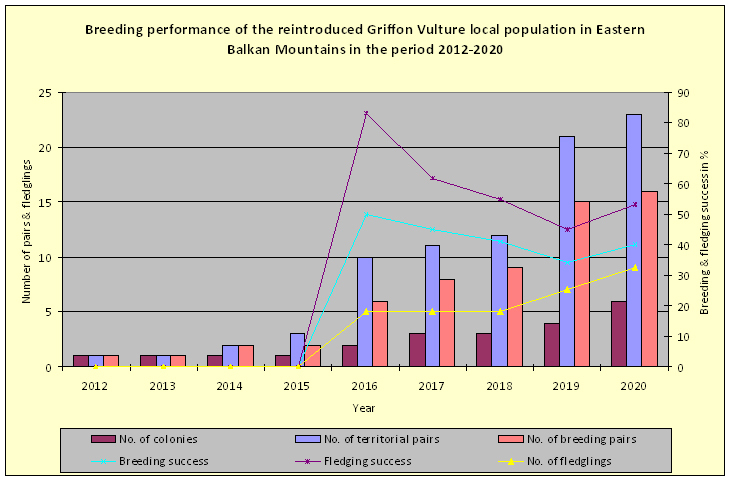
Breeding performance of the local re-introduced Griffon Vulture population in the Eastern Balkan Mountains for the period 2012-2020 - numbers of colonies, territorial pairs, breeding pairs, fledglings and relative breeding and fledging rate.

The breeding attempts of the pioneer single pairs between 2012 and 2015 were all unsuccessful, as, at that time, there were still very few mature and experienced birds. After that, the number of newly-established colonies, territorial and breeding pairs has gradually increased (Fig. 2). In the period 2016-2020, with more and more individuals reaching maturity, a total of 31-33 chicks were observed to have successfully fledged into the wild and the numbers of pairs and fledged young increases year by year. The average breeding success of the newly-restored local EBM population accounts for 0.41 fledglings/territorial pair and a fledging success of 0.61 fledglings/breeding pair between 2016 and 2020. These numbers are comparable with the productivity of 0.57 fledglings/breeding pair reported for a small Griffon Vulture colony in north-western Spain (Olea et al. 1999), yet lower than the mean breeding success of the natural population of the species in Bulgaria of 0.77 ± 0.14 and mean productivity of 0.71 ± 0.16 reported for the period 1987-2011 (Demerdzhiev et al. 2014). At the same time, Sarrazin et al. (1996) report a nesting success of 0.42 fledglings/laying pair for vultures released when older than 3 years old and 0.82 for released young and wild-born birds within a re-introduced population in the Grand Causses, Southern France (1982-1992).

With such breeding parameters, we consider the establishment phase of the re-introduced population of Griffon Vulture in the EBM successfully completed and we expect that, with time, the local birds will gain experience and the breeding parameters will further increase as seen elsewhere in other similar projects (Sarrazin et al. 1996). It should, however, be noted that the habitat conditions, climate and food source, access and availability in the EMB significantly differ from the ones in the Eastern Rhodopes, so we do not expect the re-introduced population to approach, if not reach, the breeding parameters of that particular autochthonous population.

### Number of the local population, source/sink balance

The local nucleus of Griffon Vultures in the EBM in December 2020 has stabilised at about 80 permanently fixed individuals, some 55-56 of which are tagged and thus well-recognisable, identifiable and regularly recorded. This number fluctuates with some immigration of summering or wintering birds (up to 30 individuals per season), local offspring and migrating individuals that visit the area and sojourn on the way towards their summer or wintering grounds. With a positive balance of 25 individuals for the past 5 years and an average positive balance of five individuals per year, the EBM is, therefore, considered a population source for Griffon Vulture. It is currently one of the only seven existing general areas for the species in the mainland Balkan Peninusla (Peshev et al., in prep.) and one of the five which serve as population source sites.

### Home range estimation

The average home range of the two Griffon Vultures, tracked in the EBM, is about 282 km^2^, which is less than the previous estimation of 340 km^2^ calculated in 2016 (Stoynov et al. 2018b). This is more likely due to the different methods used for calculation, rather than the genuine shrinking of the territory used by the species. However, it seems that the core of the home range comprises the two feeding sites in Kotlenska Planina SPA and SKNP, as well as several roosting and breeding sites within some 1-15 km range of the release sites (Fig. 1). Although Griffon Vultures with transmitters have been reported to make extensive movements along the Balkan Mountains and occasionally visit the Eastern Rhodopes, succeeding in undertaking some 120-200 km journeys, the 95% home range of the newly-established nucleus of the species in the EBM is relatively small compared to that of the autochthonous population in the Eastern Rhodopes - 3,220 km^2^; the re-introduced one in Kresna Gorge - 2,014 km^2^ (3,159 km^2^ together with North Macedonia) and VBNP - 4,957 km^2^ (5,058 km^2^ together with cross-border areas in Serbia) (Stoynov et al. 2018b). This relatively small home range in the EBM might provide a conservation advantage, if considered in the light of the Vulture Safe Areas concept (Peshev et al. 2018), suggesting the concentration of a large complex of management approaches, which could increase their efficiency in small, well-defined territories. On the other hand, any change in habitat parameters or quality in the area might threaten the newly-established local nucleus of Griffon Vulture and jeopardise the long-lasting local re-introduction and conservation efforts.

### Feeding on vulture restaurants and in the wild

One of the main traits of an established re-introduced population, in addition to survival and breeding performance, is the ability to forage, find and utilise natural food sources. In the case of an obligate scavenger, such as the Griffon Vulture, this certainly depends on specific characteristics of the habitat, as well as the abundance and accessiblility of carcasses of wild ungulates and livestock. A recently-published study by Arkumarev et al. (2021), provides a detailed insight into the ratio of natural (wild) food use to vulture restaurants use of the growing autochthonous Griffon Vulture population in the Eastern Rhodopes, Bulgaria. The study suggests that 80% of the foraging events occur at wild food sources and the supplementary feeding sites only play a minor role.

The situation in the EBM is the opposite, with a reported dependence on wild feedings of barely 20%. This might be attributed to: 1. lower habitat quality of the EBM area, compared to the Eastern Rhodopes, whеre the climate is warmer, providing for longer foraging flights and coherent grazing areas with more free-ranging livestock; 2. greater number of the feeding sites in the Eastern Rhodopes and larger number of vultures, flying amongst the feeding sites, thus covering a larger foraging area and encountering many more frequently accidental findings on their way; and 3. more intensive food provision in the EBM related to the local re-introduction project (about 80% of the food provided to the vulture restaurants is from local farms and some 40-50% of it would have otherwise be left on field and accessible for the vultures to locate and utilise as "wild" feedings). The dependence on wild feedings of the other large Griffon Vulture meta-populations, such as in the Gorge of Uvats and the adjacent regions in Serbia in the winter period, is also scarce and they rely very much on the vulture restaurants (Marinković et al. 2020); the same was reported also for the Eastern Rhodopes (Arkumarev et al. 2021). Only in the area of Pindus/Akarnanika, south-western Greece, the local Griffon Vultures feed entirely in the wild, as no vulture restaurants are maintained there. Free-ranging livestock is accessible in the high mountains of Pindus mountain range (i.e. National Park of Tzoumerka, Acheloos Valley, Agrafa and Meteora) in summer (Stara et al. 2016) and in Akarnanika/Messolonghi area also in winter, due to the soft Mediterranean climate (Tsiakiris et al. 2014). The local population remains, however, at a very limited number of a few pairs and is on the brink of extinction. It is not clear if this is directly due to the limited food sources or indirectly related to the frequent poisoning episodes, turning the area into an ecological trap (R. Tsiakiris, pers. comm.), that might be buffered by supplementary feeding site operation (Stoynov et al. 2018a). In any case, management practices (or lack of such) of the Griffon Vulture populations in western Greece are not applicable to the Balkan Mountains of Bulgaria due to completely different natural settings.

## Conclusions

- The establishment phase of the locally-re-introduced population of Griffon Vulture in the Eastern Balkan Mountains is complete with more than 80 permanently present individuals and some 25 territorial pairs, producing about 10 fledglings per year.
- The re-introduction cost of releases was relatively high as compared to similar re-introduction projects across Europe with great losses of released Griffon Vultures, mostly due to electrocution and probably because of emaciation of inexperienced and eventually not fully rehabilitated translocated individuals.
- The newly-established local breeding nucleus is fully integrated in the Balkan Griffon Vulture meta-population, as confirmed by the on-going permanent or temporal exchange and settling of individuals from other colonies in the Balkan Peninsula.
- The EBM produces more Griffon Vultures than eventually come and die in the area and it can, therefore, be considered as a source nucleus for the species in the national and regional (Balkan) context.
- The newly-established population mostly relies on food provided on feeding sites, but still 20% of the food is found naturally in the wild within a home range of 288 km^2^. The home range of the EBM nucleus is the smallest of all other Griffon Vulture nuclei on the Balkans. The feeding sites in the area should, therefore, be maintained and eventually integrated into the protected areas (e.g. SKNP) and Natura 2000 sites management plans and activities, while the setting up of new feeding sites should be considered in the wider nearby area in order to extend the range of the newly-established breeding nucleus.

## Figures and Tables

**Table 1.** Number of released Griffon Vultures by year and by site in the Eastern Balkan Mountains for the period 2007-2020. *Note: the releases of three birds in 2007 and five in 2009 in Kotlenska Planina SPA were experimental ones for adjustment of the practice and, thus, not considered as such from the official etsablishment phase.

Year	Eastern Balkan Mountains		**Total**
	Sinite Kamani Nature Park UTM MH43	Kotlenska Planina SPA UTM MH65	
2007	0	3*	3*
2009	0	5*	5*
2010	7	7	**14**
2011	12	11	**23**
2012	19	7	**26**
2013	8	0	**8**
2014	16	10	**26**
2015	3	4	**7**
2016	4	0	**4**
2017	11	0	**11**
2018	4	0	**4**
2019	11	4	**15**
2020	7	0	**7**
**Total**	**102**	**51**	**153**

**Table 2.** Fate of the Griffon Vultures released in the Eastern Balkan Mountains for the period 2007-2020 - status at December 2020.

Number of dead individuals in the area of release (by reason)			Number of dead individuals outside the release area (by reason)			Breeds/sojourn anywhere out of the release area	Breeds/ sojourn in the release area	Unknown fate
electrocution	poison	other	electrocution	poison	other			
26	0	8	7	1	9			
34			17			7	52	
51						59		43
**153**								

**Table 3.** Vulture drop-out during post-release/acclimatisation period in Eastern Balkan Mountains. Included are dead and recaptured individuals.

Timeframe	Number of confirmed drop-out individuals	% of total number of released (n = 153)	% of total confirmed dead and unknown (n = 94)	% from total confirmed drop-out
0 to 6 months from release	33	21.56	35.11	64.71
6 to 12 months from release	12	7.84	14.29	23.53
> 12 months	6	3.92	7.14	11.76

**Table 4.** Number and origin of the Griffon Vultures identified in the Eastern Balkan Mountains for the period 2010-2020. * From 2016 onwards, locally raised and young tagged in the nests are also included as "local". ** A total of 14 exogenous individuals, which have immigrated to EBM in previous years, but have settled in the area are considered in the "local" category since their first breeding attempt in the area.

Origin	2010	2011	2012	2013	2014	2015	2016	2017	2018	2019	2020
Eastern Balkan Mountains (local)	11	18	21	25	29	37	39*	48*	57*	54*	56*
Central Balkan National Park (CBNP)	0	0	3	4	4	5	0**	0	0	0	0
Kresna Gorge	0	1	2	2	3	1	1	0	0	0	0
Vrachanski Balkan Nature Park (VBNP)	0	0	0	1	3	3	5	3	1	0	0
Eastern Rhodopes, Bulgaria (autochthonous)	0	0	0	2	2	2	2	2	2	0	0
Serbia (autochthonous)	0	1	1	2	2	3	1	2	2	5	5
Croatia (autochthonous)	0	0	0	1	1	1	3	0	2	1	1
Israel (tagged there, but most probably of Balkan origin)	0	0	0	1	2	2	2	3	2	3	3
Unknown origin	1	2	10	18	22	27	30	34	39	59	46

**Table 5.** Breeding performance of the newly-established Griffon Vulture local population in Eastern Balkan Mountains for the period 2012-2020. The years with successful reproduction are given **in bold**.

Site	Year	# Colonies	# Territorial pairs (b)	# Breeding pairs (c)	# Fledglings (d)	Breeding success (d/b)	Fledging success (d/c)
Eastern Balkan Mountains UTM, MH65	2012	1	1	1	0	0	0
	2013	1	1	1	0	0	0
	2014	1	2	2	0	0	0
	2015	1	3	2	0	0	0
	**2016**	**2**	**10**	**6**	**5**	**0.50**	**0.83**
	**2017**	**3**	**11-12**	**8**	**5**	**0.45**	**0.62**
	**2018**	**3**	**12-14**	**9**	**5**	**0.41**	**0.55**
	**2019**	**4**	**21-23**	**15-16**	**8**	**0.34**	**0.50**
	**2020**	**5-7**	**23-25**	**16-18**	**8-10**	**0.38**	**0.53**

**Table 6.** Multi-annual dynamics of the number and mortality of Griffon Vulture in the Eastern Balkan Mountains by year for the period 2007-2020. Analysis of the population source/sink balance – the number of locally died immigrants vs. the number of the locally fledged individuals. * Simultaneously observed; ** the number of immigrant Griffon Vultures that have died in the Eastern Balkan Mountains is given in brackets.

Year	2010	2011	2012	2013	2014	2015	2016	2017	2018	2019	2020	Total
Max. # observed Griffon Vultures*	11	18	21	25	29	37	39	48	57	115	85	-
# died individuals (of them -immigrants)**	0	8	3	1	3	2	2	2	5 (1)	4 (1)	4	34 (2)
Balance – fledged locally vs. immigrants died in the area	0	0	0	0	0	0	5	5	4	5	6	25

**Table 7.** Home-range estimation.

Griffon Vulture tag	50% core area, km^2^	95% home-range, km^2^	Days of tracking
K5M (adult)	4.7	216.85	347
H1 (juvenile)	8.5	346.91	958
